# Real World—Big Data Analytics in Healthcare

**DOI:** 10.3390/ijerph191811677

**Published:** 2022-09-16

**Authors:** Daniele Piovani, Stefanos Bonovas

**Affiliations:** 1 Department of Biomedical Sciences, Humanitas University, Pieve Emanuele, 20090 Milan, Italy; 2 IRCCS Humanitas Research Hospital, Rozzano, 20089 Milan, Italy

The term Big Data is used to describe extremely large datasets that are complex, multi-dimensional, unstructured, and heterogeneous and that are accumulating rapidly and may be analyzed with appropriate informatic and statistical methodologies to reveal patterns, trends, and associations [1]. In medical and healthcare research, Big Data sources include electronic health records (EHRs), administrative or claims databases, product and disease registries, smart/wearable/self-monitoring devices, and large-scale collaborations for the collection and storage of health data and biospecimens in biobanks.

The definition of what Big Data means with respect to health research, or at least a consensus of what this term means, was proposed by the Health Directorate of the Directorate-General for Research and Innovation of the European Commission: “*Big Data in health encompasses high volume, high diversity biological, clinical, environmental, and lifestyle information collected from single individuals to large cohorts, in relation to their health and wellness status, at one or several time points*” [2].

Big Data analytics techniques and methods, such as statistical analysis, data mining, machine learning, and deep learning, have made notable progress in the recent years, and are expected to develop even further in the near future [3]. With the ever-increasing quantities of data that are digitally collected and stored within healthcare organizations, there is a growing enthusiasm in the potential applications for Big Data analytics in the fields of diagnostics, precision medicine, computerized decision support for clinicians, pharmacological research aiming to cure diseases and develop new treatments, the early detection of adverse drug reactions, cost reduction in patient care, preventive medicine, and population health research [4].

At the same time, the term Real-World Data (RWD) is commonly used to describe data derived from sources other than traditional randomized-controlled trials (RCTs). These sources may include EHRs, pragmatic clinical trials, prospective or retrospective observational studies, health insurance claims, case reports, data obtained as part of routine public health surveillance, product and disease registries, patient surveys, or other real-world sources [5].

The Association of the British Pharmaceutical Industry has defined the RWD as “*data obtained by any non-interventional methodology that describes what is happening in normal clinical practice*” [6], while according to the U.S. Food and Drug Administration, the term RWD refers to “*data relating to patient health status and/or the delivery of health care routinely collected from a variety of sources*” [7]. In the last few years, a consensus has formed that RWD offer valuable opportunities for generating robust clinical evidence (i.e., real-world evidence) regarding the use and potential benefits or harms of new therapies outside the context of RCTs [8,9].

Researchers typically understand RWD as observational data, distinct from data sourced from RCTs, and in a way similar to Big Data. Nevertheless, RWD and Big Data are not synonymous. In fact, Big Data represent a special kind of RWD, which are characterized by high volume, high velocity, high variety, high veracity, and high value (5 Vs) [10].

The promise of Big Data in healthcare depends on the ability to extract meaningful information from real-world, large-scale resources that may pave the way to scientific discoveries in the pathogenesis, diagnosis, prevention, treatment, and prognosis of diseases, and eventually revolutionize clinical medicine and public health [11,12,13]. When Big Data are analyzed with the aim of causal inference and not as a hypothesis-generating tool, special attention should be made to the very serious risk of residual confounding and a variety of biases including time-related biases [14]. Recent methodological advancements have made causal inferences from Big Data possible and, in certain cases, are highly effective in replicating the results of RCTs that are made available later [14,15]. This can be achieved successfully if a careful application of a comprehensive methodological and statistical analysis plan called “target trial emulation” is pursued [14]. The failure to correctly apply such a plan may result in Big Data yielding overly optimistic estimates of effects.

Despite these advancements, until now, Big Data analytics have not fulfilled the oversized expectations in the health sector, possibly because of several significant challenges that are summarized below [16,17,18,19]:

(a) Big Data are often unstructured, fragmented, heterogeneous, and in incompatible formats, and are thus difficult to aggregate and analyze;
(b) There are important issues regarding data security (privacy and confidentiality);
(c) A lack of data standardization, language barriers, and different terminologies;
(d) There are often problems with the accuracy and precision of data;
(e) Storage and transfers of data are associated with significant costs;
(f) Budget constraints—there is a shortage of focused and sustained funding;
(g) The awareness of Big Data analytics’ capabilities among health care professionals is rather limited;
(h) A shortage of researchers with skills in Big Data—due to the constant evolution of science and technology, professionals who collect, process, extract, or analyze data (i.e., data scientists, biostatisticians, epidemiologists, and experts in advanced analytics and artificial intelligence) need to be regularly trained and kept up-to-date;
(i) There are often issues regarding data governance and data ownership;
(j) Healthcare organizations implementing Big Data analytics as a part of their information systems need to comply with high standards and regulatory legislation.

Additionally, Sir David Cox and colleagues have methodically discussed several challenges of a statistical/epidemiological nature that arise when analyzing Big Data in healthcare [20]:

(a) The relevance of the data for the purpose of the investigation (the data’s fitness for purpose)—big datasets may not be representative of the target population, and the largeness of a dataset does not imply that the findings of the investigation (e.g., the patterns, trends, and associations) are free of bias;
(b) The need for well-established quality control and assurance procedures (data reliability)—Big Data are not collected for a specific purpose and may be subject to particular quality issues (e.g., measurement errors, missing data, errors in coding information buried in textual reports, etc.);
(c) The potential for overconfidence in the results obtained from statistical analyses of Big Data (i.e., conclusions being seriously overoptimistic) due to superficially highly precise, but potentially biased, estimates.

In conclusion, the application of Big Data in healthcare is a fast-growing field with great advances in data-generation and data-analysis methodologies. Despite the challenges outlined above, Big Data analytics have the potential for positive impacts and global implications; they are becoming increasingly important, as they enable investigations to be conducted and conclusions to be drawn that would otherwise be very difficult or even impossible. However, we should keep in mind that, when analyzing Big Data in health care research, we need to make careful use of statistical and epidemiological concepts together with an in-depth understanding of the data themselves.
